# MUDENG Expression Profiling in Cohorts and Brain Tumor Biospecimens to Evaluate Its Role in Cancer

**DOI:** 10.3389/fgene.2019.00884

**Published:** 2019-09-19

**Authors:** Juhyun Shin, Jun-Ha Choi, Seunghwa Jung, Somi Jeong, Jeongheon Oh, Do-Young Yoon, Man Hee Rhee, Jaehong Ahn, Se-Hyuk Kim, Jae-Wook Oh

**Affiliations:** ^1^Animal Resources Research Center, Konkuk University, Seoul, South Korea; ^2^Department of Stem Cell and Regenerative Biotechnology, Konkuk University, Seoul, South Korea; ^3^Department of Bioscience and Biotechnology, Konkuk Institute of Technology, Konkuk University, Seoul, South Korea; ^4^Department of Veterinary Medicine, College of Veterinary Medicine, Kyungpook National University, Daegu, South Korea; ^5^Department of Ophthalmology, Ajou University School of Medicine, Suwon, South Korea; ^6^Department of Neurosurgery, Ajou University School of Medicine, Suwon, South Korea

**Keywords:** Mu-2-related death-inducing gene, the cancer genome atlas, gene expression omnibus, patient survival, glioblastoma multiforme, cancer cell apoptosis

## Abstract

Mu-2-related death-inducing gene (MUDENG, *MuD*) has been reported to be involved in the tumor necrosis factor-related apoptosis-inducing ligand (TRAIL)-associated apoptotic pathway of glioblastoma multiforme (GBM) cells; however, its expression level, interactors, and role in tumors are yet to be discovered. To investigate whether *MuD* expression correlates with cancer progression, we analyzed The Cancer Genome Atlas (TCGA) database using UALCAN and Gene Expression Profiling Interactive Analysis (GEPIA). Differential expression of *MuD* was detected in 6 and 10 cancer types, respectively. Validation performed using data from the Gene Expression Omnibus database showed that MuD expression is downregulated in KIRC tumor and correlate with higher chance of survival. Upregulation of *MuD* expression in GBM tumors was detected through GEPIA and high MuD expression correlated with higher survival in proneural GBM, whereas the opposite was observed in classical GBM subtype. GBM biospecimens analysis shows that MuD protein level was upregulated in three of six specimens, whereas mRNA level remained relatively unaltered. Therefore, *MuD* may exert differential effects according to subtypes, and/or be subjected to post-translational regulation in GBM. Correlation analysis between GBM cohort database and experiments using GBM cell lines revealed its positive effect on regulation of protein phosphatase 2 regulatory subunit B’Epsilon (*PPP2R5E*) and son of sevenless homolog 2 (*SOS2*). STRING database analysis indicated that the components of adaptor protein complexes putatively interacted with MuD but showed no correlation in terms of survival of patients with different GBM subtypes. In summary, we analyzed the expression of *MuD* in publicly available cancer patient data sets, GBM cell lines, and biospecimens to demonstrate its potential role as a biomarker for cancer prognosis and identified its candidate interacting molecules.

## Introduction

Glioblastoma multiforme (GBM) is the most common and malignant form of primary brain tumor (Huse and Holland, 2010; Siegel et al., 2017). Despite recent advances in surgical and other therapeutic techniques, the median survival of patients with GBM is as low as 12 to 15 months (Jung et al., 2014; Ostrom et al., 2014). Aside from the conventional therapies, the selective induction of apoptosis in target cancer cells with pro-apoptotic cytokines, such as tumor necrosis factor-related apoptosis-inducing ligand (TRAIL) (Merino et al., 2007) seems promising, as this strategy exhibited low toxicity to non-cancerous cells, including brain cells, in clinical trials (Stuckey and Shah, 2013). However, the use of TRAIL is controversial because it is thought to induce apoptosis not only in cultured normal human hepatocytes but also in normal brain tissues (Jo et al., 2000). Therefore, the applicability of TRAIL for the treatment of brain cancer by combinatorial drug treatment strategies should be carefully monitored to improve its therapeutic efficacy (Stuckey and Shah, 2013).

One of the hallmarks and causes of GBM complexity is cellular heterogeneity, which poses a challenge for disease diagnosis and treatment (Friedmann-Morvinski, 2014; Inda et al., 2014). The molecular profiling of The Cancer Genome Atlas (TCGA) divides GBM into four distinctive subtypes, namely, classical, neural, proneural, and mesenchymal (Verhaak et al., 2010). Both classical and mesenchymal subtypes are aggressive in nature. Whereas the classical subtype is characterized by overexpression of epidermal growth factor receptor (EGFR), the mesenchymal subtype shows decreased neurofibromin 1 (NF1) expression and high transforming growth factor-β (TGF-β) and nuclear factor-κB (NF-κB) activities. The neural subtype is controversial because it is thought to originate from the substantial contamination of GBM samples with healthy brain tissue. Tumorigenesis of the proneural subtype starts from the frontal cortex of the cerebrum and often displays amplification of platelet-derived growth factor receptor α (PDGFRα) and mutations of isocitrate dehydrogenase 1/2 (IDH1/2) and tumor protein 53 (TP53) (Verhaak et al., 2010). Patients with proneural subtype exhibit the best prognosis but may have the worst disease outcomes in the absence of *IDH1* mutations (Phillips et al., 2006; Verhaak et al., 2010; Akan et al., 2012). Although several efforts have been directed to identify the critical driver pathways and therapeutic targets specific for each GBM subtype, very little progress has been made in this direction. A recent report revealed increased sensitivity of patients with proneural GBM to cyclin-dependent kinase 4/6 (CDK4/6) inhibitor treatment (Li et al., 2017) and significantly faster recurrence after bevacizumab treatment in patients with classic GBM (Hovinga et al., 2019), indicating the importance of careful evaluation of the subtypes before treatment.

The mu-2-related death-inducing gene (MUDENG, *MuD*), also called as the adaptor-related protein complex 5 subunit Mu 1 (AP5M1), was identified as a putative component of the fifth adaptor protein (AP) complex involved in endosomal transport (Hirst et al., 2011). *MuD* was reported to be involved in the apoptotic pathway in HeLa (Lee et al., 2008), Jurkat (Lee et al., 2008; Shin et al., 2013), and B-JAB (Lee et al., 2008) cell lines. Subsequent studies demonstrated the cleavage of MuD by active caspase-3 during TRAIL-induced apoptotic signaling (Shin et al., 2013), and the subsequent activation of the anti-apoptotic function of MuD near the BH3-interacting domain death agonist (BID) and B-cell lymphoma 2 (Bcl2) junction (Choi et al., 2016). These studies suggest a possible role for MuD in cancer cells apoptotic signaling.

In the present study, we used UALCAN and GEPIA, two web-based tools that allow in-depth analyses of RNA-sequencing data from TCGA database to assess *MuD* expression in cancer cohorts. In addition, we used the microarray data from the Gene Expression Omnibus (GEO) database to validate the selected results. We conducted an integrated analysis using 12 human brain tumor samples and GBM cancer cell lines. Furthermore, we identified the differential expression of *MuD* in tumors as well as the correlation between *MuD* expression and survival in cancer types, including specific GBM subtypes. We also identified the candidate interacting genes that were validated in GBM cell lines.

## Materials and Methods

### Data Sources

The TCGA database curated by the National Institute of Health (NIH) comprises 2.5 petabytes of data on cohorts from 33 different tumor types, including genomic profiles from microarrays and next-generation sequencing (NGS) (Tomczak et al., 2015). MET500 is a database of NGS data from 500 patients with cancers of 30 primary sites (Robinson et al., 2017). Genotype-tissue expression project (GTEx) is a database of NGS and includes the microarray data collected from nearly 1,000 individuals (Consortium, 2013). As test sets, we used data sets available from the GEO database (Clough and Barrett, 2016). E-GEOD-53757 (von Roemeling et al., 2014) and E-GEOD-22541 (Wuttig et al., 2012) was used for KIRC validation (Wuttig et al., 2012), E-GEOD-70951 (Quigley et al., 2017) and E-GEOD-10886 (Parker et al., 2009) for BRCA validation, E-GEOD-68465 (Director’s Challenge Consortium for the Molecular Classification of Lung Adenocarcinoma et al., 2008) for LUAD validation and E-GEOD-23400 (Su et al., 2011) for ESCA.

### Statistical Analysis

Analysis of the TCGA data was carried out with UALCAN (RRID: SCR_015827) (Chandrashekar et al., 2017), and GEPIA (Tang et al., 2017). The differential expression of *MuD* and patient survival were analyzed with PanCan analysis and expression on box plot, respectively. Cox proportional hazard analysis was performed with GBM-BioDP provided at the Glioblastoma Bio Discovery Portal (https://gbm-biodp.nci.nih.gov/) (Celiku et al., 2014). Patients were divided based on the diagnosed GBM subtype and further stratified into four quartiles as per *MuD* expression level. For each group, a Cox proportional hazard model was used to plot the survival of the patients from the first quartile versus those from the fourth quartile using age and O-6-methylguanine-DNA methyltransferase (*MGMT*) methylation status as covariates. The microarray data from E-GEOD-53757 and E-GEOD-070951 processed with MAS.5 and limma (RRID : SCR_010943) in R, respectively, were visualized as heat maps using ClustVis (https://biit.cs.ut.ee/clustvis/) (Metsalu and Vilo, 2015). Heat maps were row-centered and unit variance scaling was applied for rows. Principal components were calculated using the NIPALS PCA method included in pcaMethods R package, and heatmaps were plotted using heatmap R package (version 0.7.7). Differential expression and survival plots were plotted using survminer R package (version 0.4.4) after processing with limma package and z-score (value-mean normal value/normal SD) calculated by R. Student’s *t*-test was used to analyze differences between groups in the real-time quantitative polymerase chain reaction (RT-qPCR) and immunoblot data.

### Sample Collection From Human Brain Tumors

This study was approved by the Konkuk University Institutional Review Board (IRB; 7001355-124 201512-E-041), and all patients signed IRB-approved consent forms. The biospecimens used in the present study were provided by the Ajou Human Bio-Resource Bank (Suwon, Korea), a member of the National Biobank of Korea, supported by the Ministry of Health and Welfare. All samples derived from the National Biobank of Korea were obtained with informed consent under institutional review board-approved protocols. We obtained 12 tissues from the following patients (**Table 1**): 10 patients diagnosed with glioblastoma grade IV, and healthy tissues of six of these patients; one patient diagnosed with oligodendroglioma grade II; and one patient diagnosed with ependymoma grade II. Samples were stored below −80°C until nucleic acid and protein extraction.

**Table 1:** Differential *MuD* expression and survival correlation revealed by UALCAN and GEPIA.

	Project name	UALCAN	GEPIA	GR(U)	GRG)	S (U)	S (G)
BRCA	Breast invasive carcinoma	N (n = 114)	N (n = 291)	NS	NS	C	No
		T (n = 1094)	T (n = 1085)			(HL)	
CHOL	Cholengiocarcinoma	N (n = 9)	N (n = 9)	Up	NS	No	No
		T (n = 36)	T (n = 36)				
COAD	Colon adenocarcinoma	N (n = 41)	N (n = 349)	Down	NS	No	No
		T (n = 286)	T (n = 275)				
DLBC	Lymphoid neoplasm diffuse large B-cell lymphoma	–	N (n = 337)	–	Up	No	No
			T (n = 47)				
ESCA	Esophageal carcinoma	N (n = 11)	N (n = 286)	Up	Up	No	No
		T (n = 184)	T (n = 182)				
GBM	Glioblastoma multiforme	N (n = 5)	N (n = 207)	–	Up	No	No
		T (n = 156)	T (n = 163)				
KICH	Kidney chromophobe	N (n = 25)	N (n = 53)	NS	Up	No	No
		T (n = 67)	T (n = 66)				
KIRC	Kidney renal clear cell carcinoma	N (n = 72)	N (n = 100)	Down	Down	.C	C
		T (n = 533)	T (n = 523)			(HH)	(HH)
KIRP	Kidney renal papillary cell carcinoma	N (n = 32)	N (n = 60)	Down	NS	No	No
		T (n = 290)	T (n = 286)				
LGG	Lower-grade glioma	N (n = 248)	N (n = 207)	NS	Up	No	No
		T (n = 265)	T (n = 518)				
LUAD	Lung adenocarcinoma	N (n = 29)	N (n = 347)	NS	NS	C	No
		T (n = 519)	T (n = 483)			(HL)	
OV	Ovarian serous cystadenocarcinoma	–	N (n = 88)	–	Up	No	No
			T (n = 426)				
PAAD	Pancreatic Adenocarcinoma	N (n = 4)	N (n = 171)	NS	Up	No	No
		T (n = 178)	T (n = 179)				
READ	Rectum adenocarcinoma	N (n = 11)	N (n = 92)	Down	NS	No	No
		T (n = 166)	T (n = 318)				
STAD	Stomach adenocarcinoma	N (n = 34)	N (n = 34)	NS	Up	No	No
		T (n = 415)	T (n = 415)				
THYM	Thymoma	N (n = 2)	N (n = 339)	NS	Up	No	No
		T (n = 120)	T (n = 118)				

Abbreviations of cancer types based on TCGA are given in the left column with project name. UALCAN and GEPIA sample sizes for normal (N) and tumor (T) tissues are indicated for comparison as available or excluded if normal tissue data were unavailable. Gene regulation (GR) upon detection was listed as Up or Down as relevant and according to UALCAN (U) and GEPIA (G). Survival (S) correlation with expression is listed as correlating (C). High expression = Low survival chance (HL) and High expression = High survival chance (HH).

### Cell Lines and Cell Culture

The U251-MG cell line (NCI-DTP Cat U-251, RRID: CVCL 0021) was obtained from Dr Benveniste EN (University of Alabama at Birmingham, Birmingham, AL, USA). The U251-MG *MuD* knock-out (KO) line β18 was generated using clustered regularly interspaced short palindromic repeats (CRISPR)/Cas9 plasmid SpCas9-2A-puro (PX459) V 2.0 provided by Feng Zhang (RRID: Addgene_6288) and single guide RNA 5′-ACACTAATTAGTGGCGGACG-3′ designed with CRISPR DESIGN (http://crispr.mit.edu/). U251-MG cells stably expressing (SE) GFP alone (C1) and GFP-MuD (C1MuD) were generated by transfection using Lipofectamine 2000 and subsequently selected with G418 sulfate (200 µg/ml; Invitrogen, USA). The cells were maintained in minimum essential media (MEM; Gibco, USA) supplemented with 10% fetal bovine serum, 100 U/ml penicillin, and 100 µg/ml streptomycin (Welgene, Daegu, Korea). All cell lines were cultivated at 37°C in a humid 5% CO_2_ chamber and subcultured every 3 days after they reached 80% to 90% confluency. The cells were not subcultured beyond 20 passages.

### RT-qPCR Analysis

RNA and proteins were extracted using Nucleospin^®^ RNA/Protein (Macherey-Nagel, BMS, Korea) according to the manufacturer’s instructions. cDNA was synthesized using AccuPower^®^ RT/PCR PreMix (Bioneer, Korea). qPCR was performed with SYBR qPCR Mix (CellSafe, Yongin, Korea) on a CFX96 Real-Time System (Bio-Rad, BMS, Korea). Data were analyzed with the Pfaffl method (Pfaffl, 2001) using glyceraldehyde 3-phosphate dehydrogenase (*GAPDH*) gene as reference.

### Immunoblot Analysis

Sample lysates were subjected to sodium dodecyl sulfate polyacrylamide gel electrophoresis (SDS-PAGE) analysis, and the separated bands were transferred onto polyvinylidene difluoride membranes. The membranes were blocked with 5% non-fat milk and incubated with MuD monoclonal antibody (Wagley et al., 2013) at 4°C overnight. The blots were subsequently incubated with a horseradish peroxidase (HRP)-labeled anti-human IgG at room temperature (15–20°C) for 2 h. The immunoreactive bands were detected with an enhanced chemiluminescence substrate (Dogen, Seoul, Korea), and band intensities were measured with ImageJ. All primers and antibodies used are listed in **Supplementary Table 1**.

## Results

### Differential Expression of *MuD* in 14 Cancers Types

Our previous study suggested the involvement of *MuD* gene in the apoptotic pathway of the GBM cell line U251-MG induced by TRAIL (Choi et al., 2016). To investigate the role of *MuD* in cancer, we analyzed *MuD* expression data from the TCGA database (Cancer Genome Atlas Research et al., 2013), which included 35 different cancer types with normalized RNA expression for 33,096 cases as of December 2018. The analysis was performed with UALCAN, a web-based tool that facilitates in-depth analysis of the TCGA, and MET500 transcriptome databases (Chandrashekar et al., 2017) and GEPIA, which use the TCGA and GTEx projects databases to compare gene expression between tumor and normal tissues (Tang et al., 2017). Differential regulation of *MuD* gene expression was detected in six cancer types with UALCAN and 10 cancer types using GEPIA for a total of 14 cancer types. In most cases, the sample size was larger in the GEPIA database than in UALCAN. *MuD* expression in tumors was upregulated as compared with that in normal tissues in 9 of the 10 cancer types identified by GEPIA versus only two of the eight cancer types identified with UALCAN. *MuD* expression was downregulated only in kidney renal clear cell carcinoma (KIRC) tumor tissues, as per GEPIA analysis, but three additional cancer types were identified with UALCAN. Both of these tools detected *MuD* upregulation in the tumor tissues from patients with esophageal carcinoma (ESCA) and downregulation in patients with KIRC (**Table 1**). To validate if tumor purity of the TCGA tumors might affect the outcome, consensus measurement of purity (CPE) as previously described (Aran et al., 2015) was used to select GBM and KIRC-TCGA tumor >0.9 and 0.7 based on tumor purity distribution of the samples. Results consent with UALCAN results, suggesting that divergent result from UALCAN and GEPIA is not due to TCGA tumor quality (**Supplementary Figure 1**).

### Validation Using Test Data Sets

**Figure 1:**
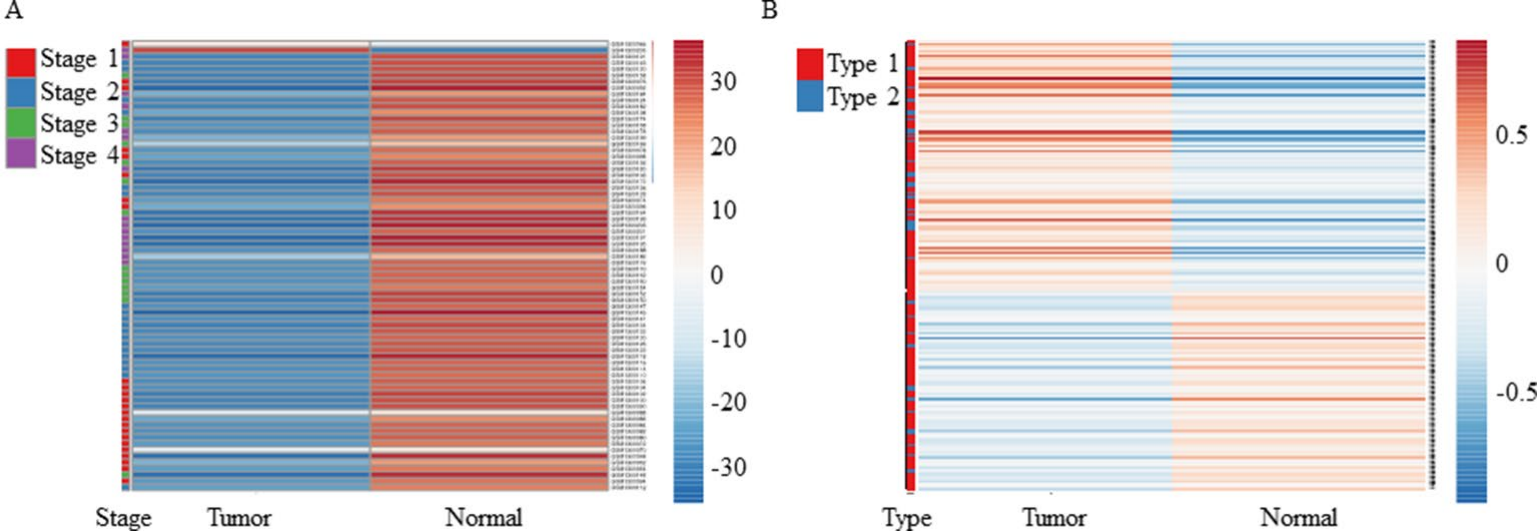
Heatmap showing the expression of *MuD* from microarray data. *MuD* expression in kidney renal clear cell carcinoma **(A)** and adjacent kidney tissue as well as in breast cancer **(B)** and adjacent breast tissue. Clustering was performed with ClustVis using NIPALS PCA method. Red–white–blue scale was used to depict the normalized expression level. Red, blue, green, and violet color bars were used in clear cell renal carcinoma to represent classified stages, whereas red and blue bars were used in breast cancer to represent the diagnosis type.

As mentioned above, both tools revealed downregulation of *MuD* in KIRC tumor tissues but failed to detect any significant dysregulation in breast invasive carcinoma. To validate these findings, we selected two test sets from EMBL-EBI ArrayExpress database. Microarray data of patients with renal clear cell carcinoma (E-GEOD-53757) and breast adenocarcinoma (E-GEOD-70951) as control were analyzed. Except for two tumor samples, all tissues from patients with renal cancer showed significantly downregulated *MuD* expression levels relative to the matched normal tissues (**Figure 1A**). In contrast, *MuD* expression in the control data set was differently regulated in tumor tissue as compared with normal tissue (**Figure 1B**), indicative of the absence of any correlation between *MuD* expression and tumor identity. According to both portal, ESCA was up-regulated in tumor. This was validated using E-GEOD-23400 (**Supplementary Figure 2**).

### *MuD* Expression Patterns Correlated With Survival in Three Different Cancer Types Based on TCGA Database

**Figure 2:**
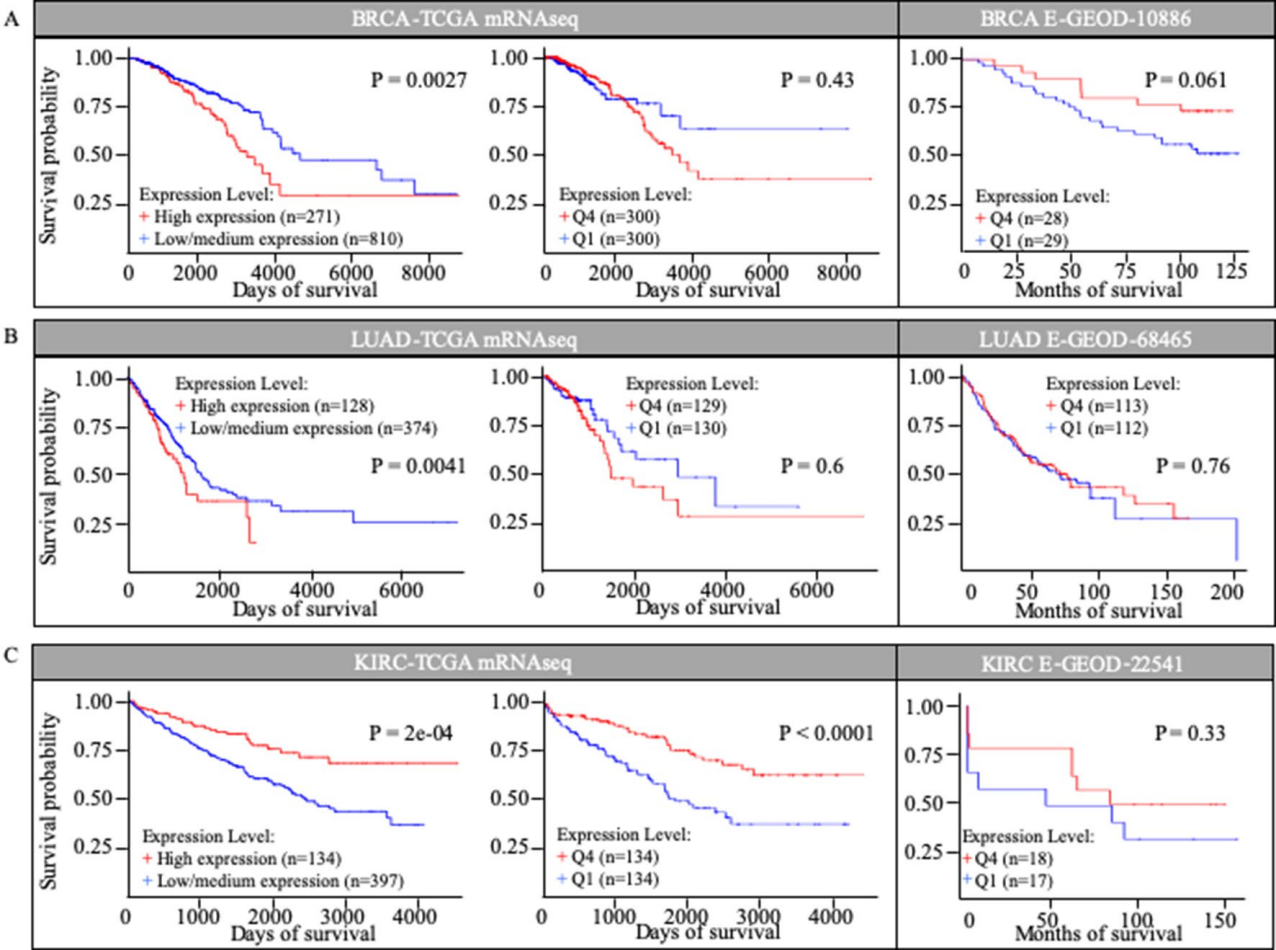
Kaplan-Meier survival curves in TCGA cohorts and E-GEOD data sets. Survival curves based on MuD expression was plotted for breast invasive carcinoma (BRIA) **(A)**, lung adenocarcinoma (LUAD) **(B)**, and kidney renal clear cell carcinoma (KIRC) **(C)**.

We investigated the correlation between *MuD* expression and patient survival in selected TCGA cohorts. Kaplan-Meier survival curves were generated using UALCAN for three cancer types based on the information in the TCGA database (**Figure 2**). UALCAN use statistical analysis that divided patients into two groups, comparing the higher quartile to the rest based on *MuD* expression. Among patients with invasive breast cancer (BRCA), those with high *MuD* expression (n = 271) revealed significantly lower survival (*p* < 0.005) than the controls (n = 810). In LUAD, a similar pattern was observed in the cohort characterized with high MuD expression (n = 128) relative to the corresponding controls (n = 374) (*p* < 0.05). In KIRC, survival was significantly lower in the cohort with high MuD expression (n = 134) (*p* < 0.0001) than in controls (n = 397) (**Figures 2A, B, and C** left plot). However, when equal number of samples were used to analyze survival chance, both BRCA and LUAD-TGCA lost their significance (p > 0.1) (**Figures 2A, B, and C**, middle plot). Validation using E-GEOD data shows that BRCA and LUAD outcomes from TCGA database are divergent from testing data set. Although KIRC data from E-GEOD-22514 lack vital status needed to analyze survival, this can be interfered from data of months free of tumor and total follow-up months as previously tested(Chang et al., 2018). Although *p* value was high due to the small numbers of samples, higher MuD expression correlated with higher survival, as it was for TCGA data (**Figure 2C**). Therefore, validation shows that although UALCAN is more sensitive in detecting potential correlation between expression and survival, GEPIA give a more robust outcome, possibly due to the fact that UALCAN analyze survival with unbalanced numbers of samples.

### *MuD* Expression Pattern Correlated With High Survival in Proneural GBM Subtypes and Low Survival in Classical GBM Subtypes

**Figure 3:**
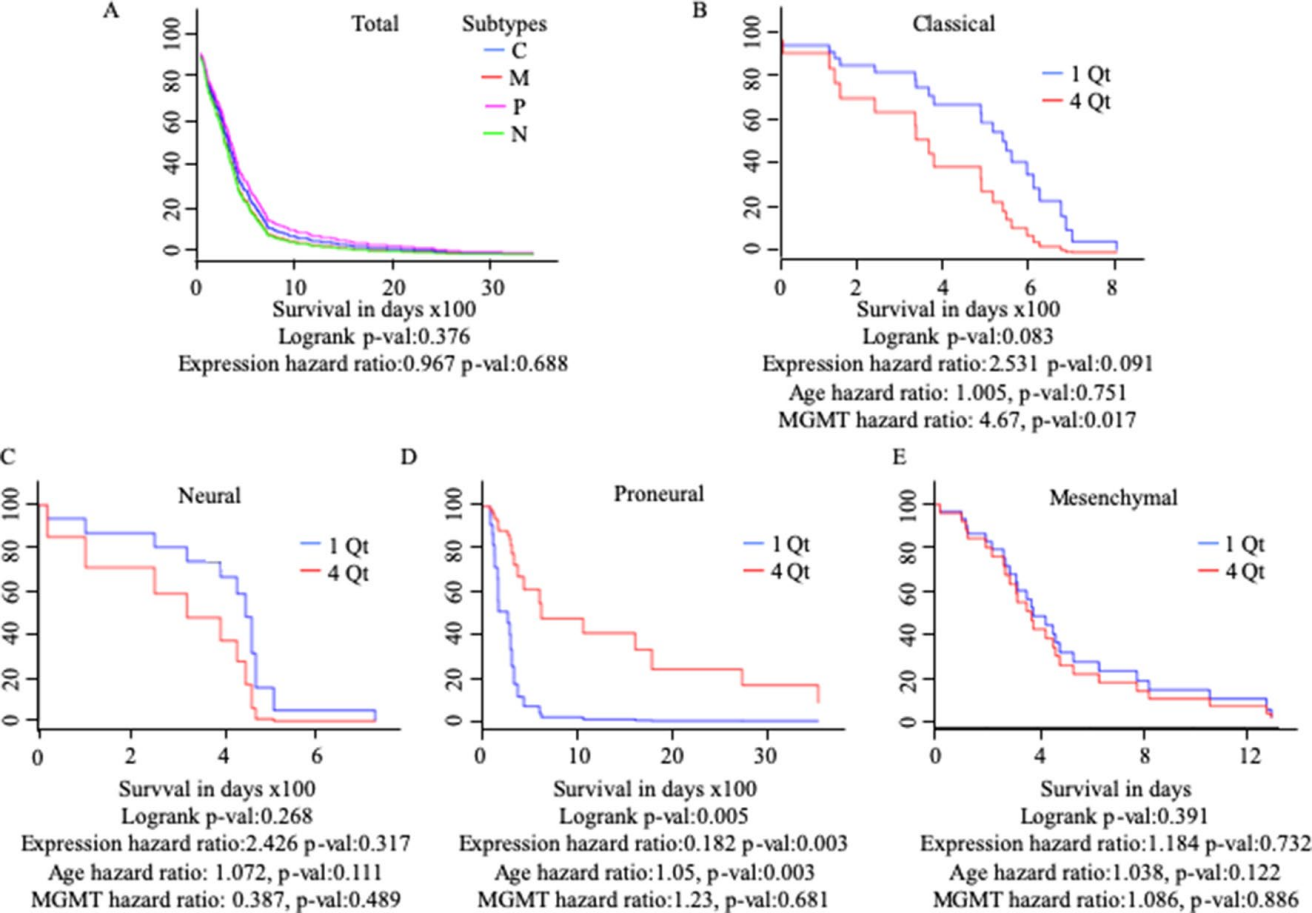
Cox proportional analysis of 422 patients with GBM from the TCGA database divided into subtypes based on *MuD* expression level, as analyzed with GBM Bio Discovery Portal for total GBM **(A)**, Classical subtype **(B)**, Neural subtype **(C)**, Proneural subtype **(D)** and Mesenchymal subtype **(E)**. Patients were ranked into four quartiles based on *MuD* expression level. The survival rate of the patients from the first quartile with the lowest ranked *MuD* expression was compared to that of the patients from the fourth quartile. Age and *MGMT* promoter methylation status were used as covariates. C, classical; M, mesenchymal; P, proneural; N, neural; QT, quartile.

Differential gene regulation in GBM tissues was detected with GEPIA but not UALCAN (**Table 1** and **Supplementary Figure 3**). As patients with GBM can be divided into subtypes with distinct molecular characteristics (Verhaak et al., 2010), we examined the survival of patients with different subtypes based on *MuD* expression. We analyzed 422 GBM samples available from TCGA. Patients were divided based on GBM subtype and further stratified into four quartiles based on *MuD* expression levels. For each subgroup, the Cox proportional hazard model was used to plot the survival of patients in the first quartile versus those in the fourth quartile, with age and *MGMT* methylation status as covariates (**Figure 3**). Interestingly, patients with proneural GBM from the fourth quartile showed significantly higher survival (*p* < 0.005), with a log-rank *p* < 0.005 and a hazard ratio (HR) value less than 1 (HR = 0.182). The age HR was slightly higher than 1 (HR = 1.05) and a high significance was observed (*p* < 0.005), suggesting that age may have a minor negative impact on the survival of patients with proneural GBM. The opposite results were observed in patients with classical subtype GBM, wherein the expression HR was higher than 1 (HR = 2.531) and a moderate significance was reported (*p* < 0.1). Interestingly, MGMT methylation was significantly more beneficial (HR = 4.67, *p* < 0.05) than age (HR = 1.005) in this group.

### *MuD* Expression Correlated With That of *EXOC5*, *PPP2R2E*, and *SOS2* and *MuD* Overexpression Upregulated *PPP2R5E* and *SOS2*

**Figure 4:**
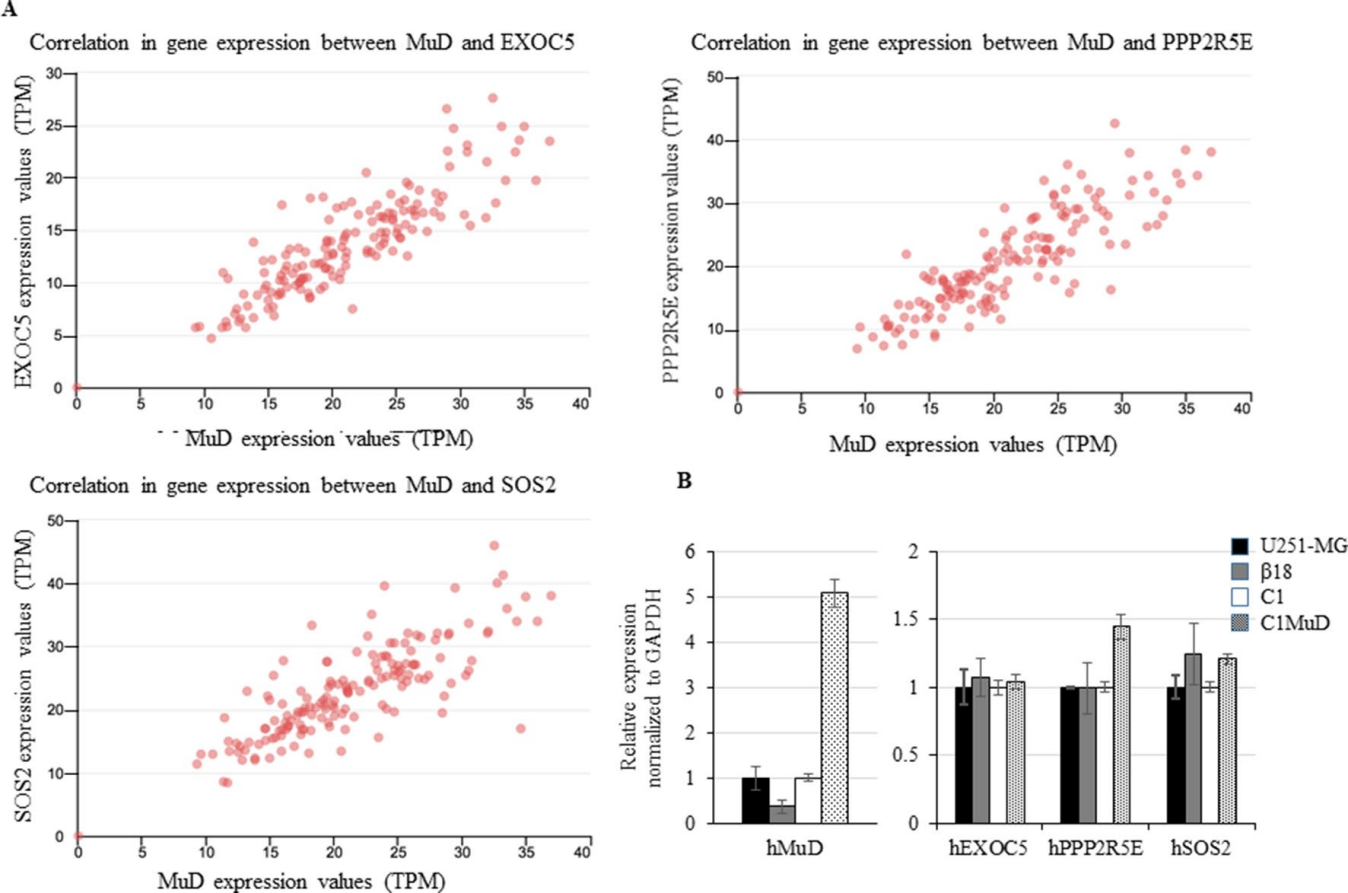
Correlation between *EXOC5*, *PPP2R5E*, and *SOS2* expression levels and *MuD* levels. Expression in GBM tissues from TCGA database **(A)** and expression of *MuD*, *EXOC5*, *PPP2R5E*, and *SOS2* in the GBM *MuD* KO line β18 and *MuD* overexpression line C1MuD as compared with that in their respective controls **(B)**.

We investigated the tumor-related genes in GBM tissues that showed correlation with *MuD* expression. Based on UALCAN results, we identified exocyst complex component 5 (*EXOC5*), protein phosphatase 2 regulatory subunit B’Epsilon (*PPP2R5E*), and son of sevenless homolog 2 (*SOS2*) to exhibit high correlations with *MuD* expression in GBM tumors from TCGA patients (Pearson’s correlation coefficient > 0.79) (**Figure 4A**). Patient survival data based on *EXOC5*, *PPP2R5E*, and *SOS2* expression levels were available in the TCGA-GBM database, and the analysis with Cox proportional hazard model revealed that the high expression levels of these genes were associated with high survival in patients with proneural GBM at a log-rank *P* value cutoff of 0.05 (*PP2R5E* and *SOS2*) or close to 0.05 (*EXOC5*) (**Supplementary Figure 4**). Correlation in BRCA, KIRC, LUAD, ESCA, and CHOL were also displayed as a table for the upmost linked 10 genes (**Supplementary Table 2**). EXOC5 shows high correlation with MuD in all subtypes except KIRC. To further investigate the correlation between *MuD* and these genes, we used a GBM cell line, U251-MG, a CRISPR-Cas9-generated *MuD* KO line β18, a plasmid transfection line containing pEGFP-C1 (C1), and a line SE *MuD* following MuD-GFP-C1 transfection (C1MuD). Although *MuD* KO failed to affect the expression of *EXOC5*, *PPO2R5E*, and *SOS2*, *MuD* stable expression increased *PPP2R5E* and *SOS2* expression levels to some extent (**Figure 4B**). The expression of these genes was also investigated in the biospecimens mentioned below, but the Pearson’s correlation coefficient was insignificant, probably owing to the small sample number (**Supplementary Figure 5**).

### Correlation Analysis Suggested the Possible Interactions Between *MuD* and Other Proteins That Affected Prognosis in Patients With GBM

**Figure 5:**
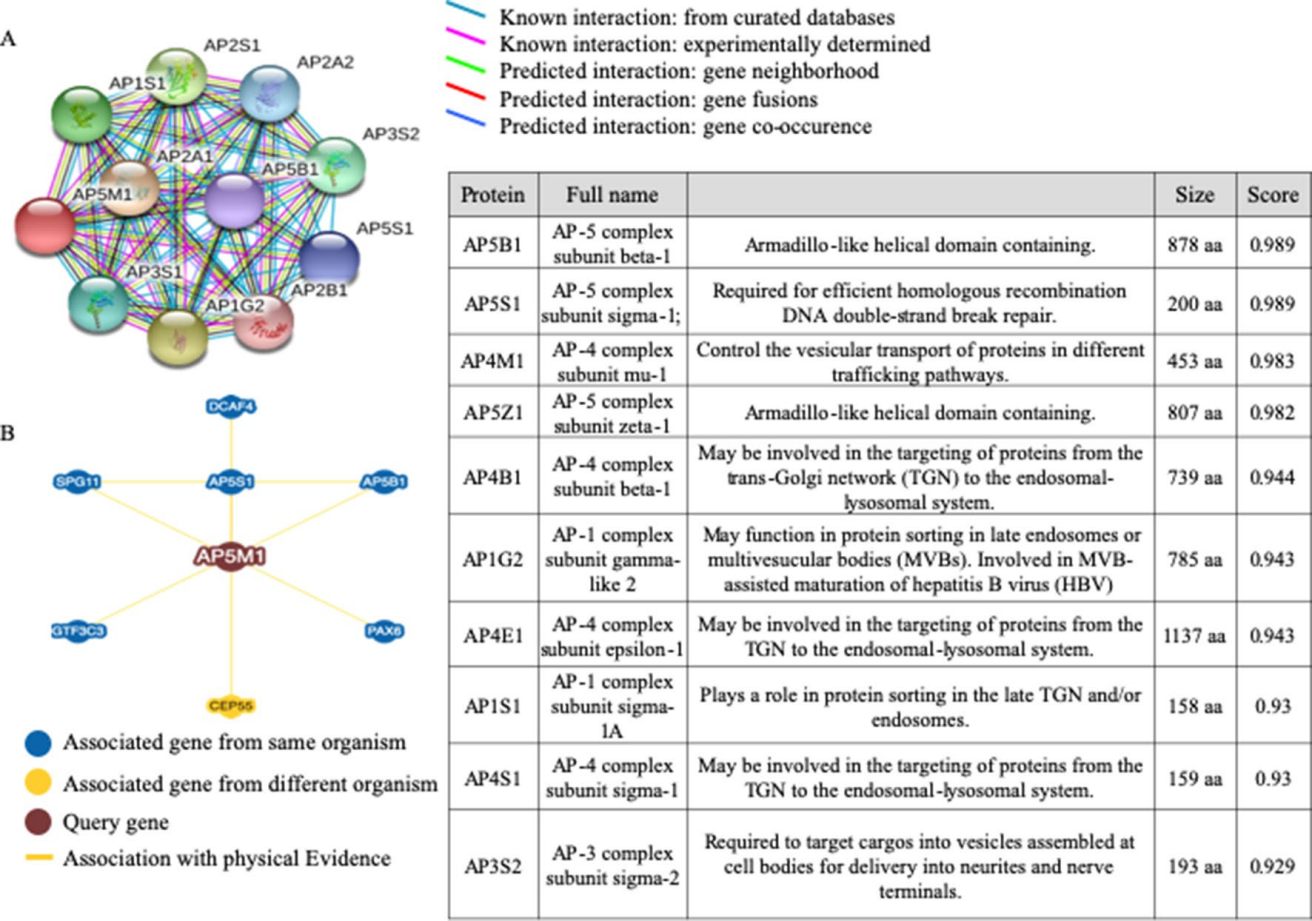
Analysis of candidate proteins interacting with MuD. STRING analysis showing interactions between MuD (AP5M1) and other adaptor protein complex subunits **(A)**. BioGRID analysis revealing the additional putative MuD interacting proteins **(B)**.

To examine the network of proteins that potentially interact with MuD, we used STRING (Szklarczyk et al., 2017) (**Figure 5**). The identified interactors were found to be other components of the AP complexes. Survival data were unavailable for the identified proteins, AP5B1, AP5S1, AP5Z1 (other putative components of the fifth AP complex) and AP4B1 and AP4S1 (components of the fourth AP complex). The components of the fourth AP complex showed opposite patterns in terms of survival and MuD expression, as the first quartile patients with AP4M1 had higher chances of survival. Another component of the fourth complex, AP4E1 showed a similar pattern with MuD in proneural subclass but revealed a different pattern in the mesenchymal subclass, wherein survival chance was highly correlated with the fourth quartile patients of AP4E1. Components of the first AP complex (AP1G2 and AP1S1) as well as the third (AP3S2) and fourth (AP4S1) complexes showed no correlation between expression and survival in patients with proneural GBM (**Supplementary Figure 6**). This discrepancy among components of similar complexes suggests the possibility of additional roles of MuD aside from its involvement with the component of AP5. BioGrid (Chatr-Aryamontri et al., 2017) shows that besides other AP complex subunits, DDB1 and CUL4-associated factor 4 (DCAF4), spatacsin vesicle trafficking-associated (SPG11), general transcription factor IIIC subunit 3 (GTF3C3), and paired box protein PAX-6 (PAX6) are candidate protein interactors for MuD in human cells. Further investigation is warranted to validate the interactions of these components with MuD.

### Expression Analysis Revealed Putative Post-Translational Regulation of *MuD*

**Figure 6:**
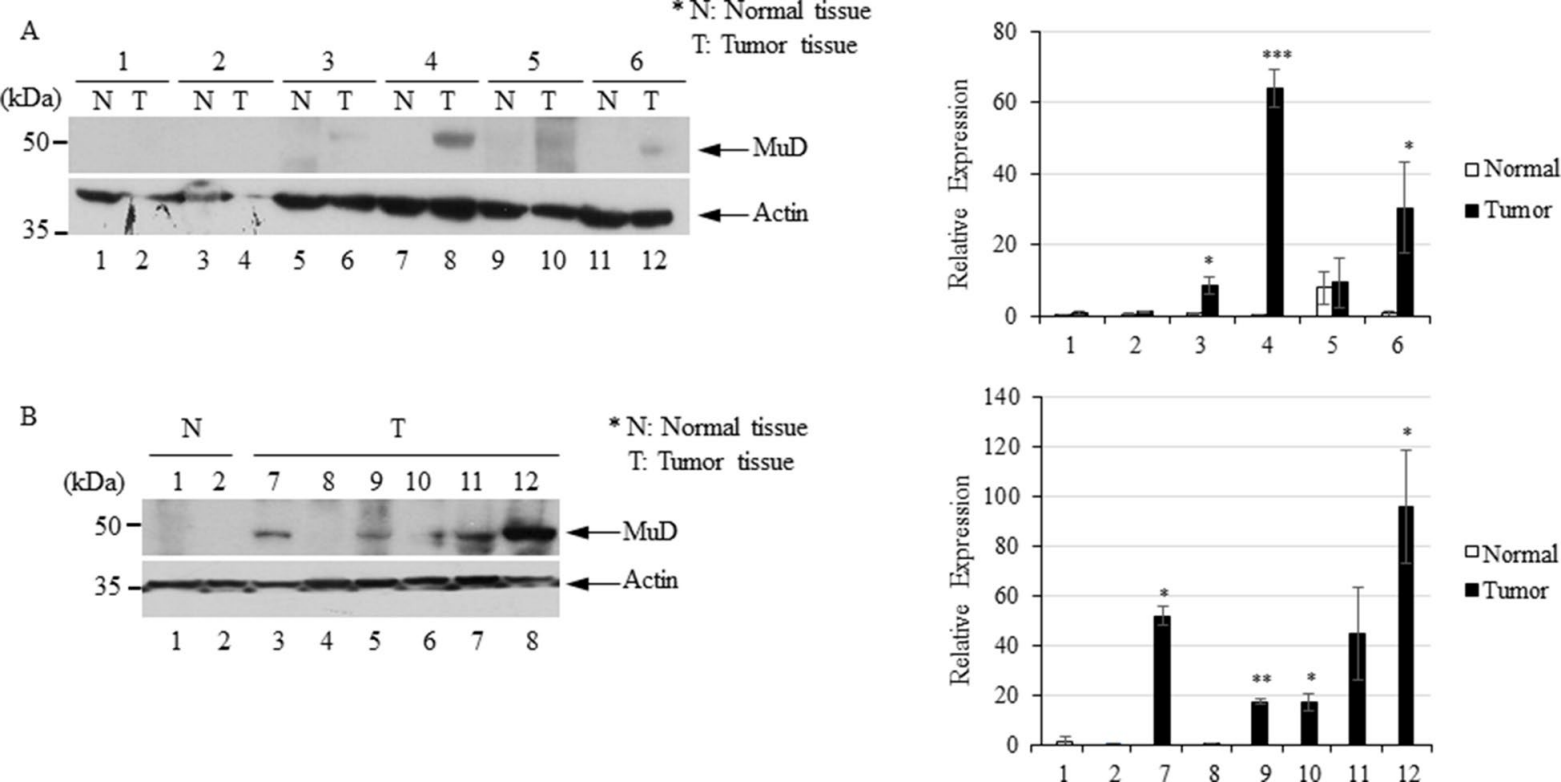
Immunoblot analysis of MuD expression in six pairs of GBM **(A)** and GBM and astrocytoma biospecimens **(B)**. N, normal tissue; T, tumor tissue *p < 0.01, **p < 0.005, ***p < 0.001.

**Figure 7:**
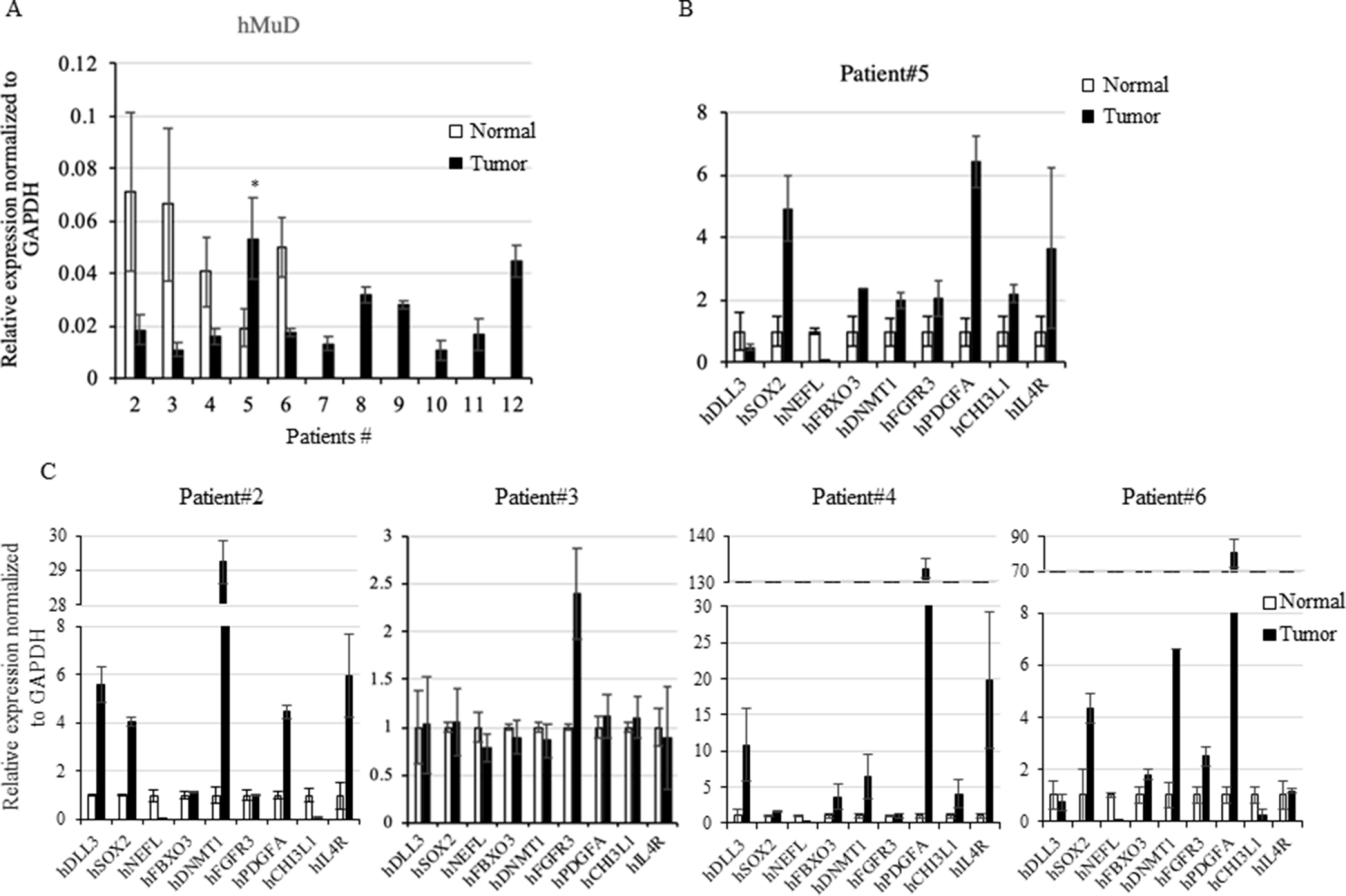
Rt-qPCR analysis of *MuD* and subtypes marker genes expression. Expression of *MuD* mRNA analyzed with RT-qPCR in patient tissues across GBM and astrocytoma brain tumor tissues **(A)**. Expression of subtype markers in tissues from patient 5 **(B)** and other patients expression of subtype markers **(C)**. mRNA expression was normalized to *GAPDH* levels (*p < 0.05).

To investigate MuD expression patterns in human brain tumor tissues, we isolated RNA and protein from six tumor biospecimens (four GBMs, one oligodendroglioma, and one ependymoma) and matched normal tissues (**Table 2**). Of these, four pairs exhibited MuD upregulation in the tumors as compared with that in the matched normal tissues, with high significance in three samples (**Figure 6A**, lane 3, 4, and 6). Two additional GBM samples (**Figure 4B**, lanes 7 and 9) and oligodendroglioma and ependymoma samples (**Figure 6B**, lanes 11, and 12) showed higher MuD expression than normal tissues. These findings imply that MuD expression may be upregulated in brain tumors, at least in GBM and other glioma tissues. Interestingly, neither MuD mRNA and protein levels nor subtypes showed any significant correlation, as MuD was only upregulated in patient 5 (**Figure 7A**). All but one (NEFL) subtype marker showed upregulation in patient 5 (**Figure 7B**) as compared with that in the other patients (**Figure 7C**), suggestive of the possible failure of gene downregulation in the tumor from this patient. As MuD protein expression was upregulated in at least four patients, there is a possibility of putative post-translational control of MuD in brain tumors without ruling out the chances of contamination from neighboring tissues.

**Table 2:** Clinical characteristics of patients with brain tumor in this study. GB, glioblastoma; ODG, oligodendroglioma; ED, ependymoma.

Patients	Tissue	Tissue bcode	Diagnosis	WHO grade	Age	Gender	Tissue	Comment
1	ANC-13-0005	25502083	GB	4	81–85	Male	Pair	High necrosis rate
2	ANC-13-0028	25502722	GB	4	81–85	Female	Pair	–
3	ANC-13-0018	25680967	GB	4	61–65	Female	Pair	–
4	ANC-13-0019	25371685	GB	4	61–65	Male	Pair	–
5	ANC-13-0027	25333994	GB	4	71–75	Female	Pair	–
6	ANC-13-0052	25072638	GB	4	56–60	Female	Pair	–
7	ANC-13-0014	25502189	GB	4	71–75	Male	Tumor	–
8	ANC-13-0022	25502458	GB	4	76–80	Female	Tumor	–
9	ANC-14-0032	25756981	GB	4	41–45	Male	Tumor	–
10	ANC-15-0009	25227244	GB	4	56–60	Male	Tumor	–
11	ANC-14-0041	25537678	ODG	2	36–40	Female	Tumor	Low tumor cells percentage
12	ANC-15-0061	25022741	ED	2	51–55	Female	Tumor	–

## Discussion

*MuD* was identified in a screening approach aimed to reveal any novel genes involved in Fas-mediated apoptosis (Kawasaki and Taira, 2002). *MuD* plays a specific role in several cancer cells (Lee et al., 2008; Choi et al., 2016), and is thought to exert its functions through apoptotic signaling, considering its cleavage by active caspase-3 upon TRAIL stimulation (Shin et al., 2013). However, the detailed roles of *MuD* in tumors remain to be elucidated. Herein, our findings based on the careful analyses of both metadata and data from patients with brain tumor suggest that any alterations in *MuD* expression could be associated with tumor progression and survival in selected cancer types.

We used two web-based portals to evaluate the potential role of *MuD* in cancer. Although the key data source for both UALCAN and GEPIA data is TCGA, only two cancer types were detected in both portals, including KRIC and ESCA. This discrepancy between the two portals may be related to the different data processing method or owing to the use of additional data from MET500 and GTEx projects, respectively. Although more cancer types were found to exhibit *MuD* upregulation in tumors using GTEx, UALCAN was more prone to detect cancer types and survival correlation with *MuD* downregulation. This result may be partially attributed to the fact that GTEx projects collected more samples, including healthy tissue data, for most cancer types, whereas both MET500 and GTEx projects had no data on patient survival. As a consequence, drastic changes in *p* and log-rank values were reported. Nevertheless, we identified two cancer types, wherein *MuD* may exhibit a role in tumor development and serve as a potential biomarker. Our results also demonstrate that although large-scale data analysis may be useful in finding novel oncogenes and new biomarkers, the data should be carefully validated.

In a previous study, we reported the anti-apoptotic function of *MuD* in GBM cell lines (Choi et al., 2016) and investigated the correlation between *MuD* expression and cancer progression. We failed to observe any correlation between *MuD* expression and overall survival in patients with GBM. However, GEPIA analysis suggested the upregulation of *MuD* in GBM tumors, and MuD protein levels were markedly upregulated in human brain tumor tissues, including 10 GBMs. *MuD* mRNA level from the same tissues showed no correlation except one at *p* < 0.05. Although this observation may be related to the small number of biospecimens investigated, additional regulation of *MuD* may occur at the post-translational level. This hypothesis is consistent with our previous finding that MuD was downregulated following TRAIL stimulation without any alteration in the *MuD* mRNA level (Choi et al., 2016).

Proneural GBM differs from other GBM subgroups with respect to gene expression patterns and responses to drug treatment (Chen and Xu, 2016). The proneural GBM cohort showed significantly improved prognosis as compared with patients with other subtypes (Verhaak et al., 2010) but failed to respond to immunotherapy as efficiently as the mesenchymal GBM cohort, presumably owing to TGF-R2 deficiency (Beier et al., 2012). Our findings showed that higher MuD expression levels were associated with prolonged survival in patients with proneural GBM; however, this correlation could not be extended to all patients with GBM. Although we failed to notice any correlation between GBM subtypes and MuD expression in our biospecimens, probably owing to the small sample size and the markers investigated, *MuD* expression might exert differential effects based on GBM subtypes and *MuD* may serve as a potential target gene specifically for the treatment of proneural subtype.

Analyses of MuD protein level, localization, and interactions with other putative proteins suggest its importance as a component of the putative AP5 complex (Hirst et al., 2011). Clathrin AP complexes play crucial roles in protein sorting in diverse post-Golgi pathways and are involved in endocytosis (McMahon and Boucrot, 2011). In particular, the AP1 complex is involved in trafficking between the trans-Golgi network (TGN) and endosomes (Hirst et al., 2012), AP2 is associated with endocytosis (McMahon and Boucrot, 2011), and AP3 mediates trafficking between the TGN/endosome and the vacuole/lysozyme system (Dell’Angelica, 2009). AP4 was thought to play a role in vacuolar sorting in plant cells (Fuji et al., 2016) and interact with Tepsin (Frazier et al., 2016). AP complexes are involved in several diseases, including X-linked mental retardation (Tarpey et al., 2006), Alzheimer’s disease (Burgos et al., 2010), and Hermansky–Pudlak syndrome (Dell’Angelica et al., 1999). Whereas these complexes showed no correlation with MuD, the two putative partner proteins of MuD, AP4M1, and AP4E1, are components of the AP4 complex and showed correlation with cancer prognosis. A recent report showed that AP4 promotes oncogenic phenotype and drug resistance in breast cancer through the regulation of a novel oncogene, lysosomal-associated protein transmembrane-4 beta (*LAPTM4B*) (Wang et al., 2018), and induces prostate cancer proliferation though l-plastin regulation (Chen et al., 2017). These studies suggest that AP complexes may play a role in cancer cell proliferation.

Aside from its role as a component of the AP5 complex, MuD is involved in cancer pathogenesis (Merino et al., 2007; Johnstone et al., 2008; Cullen and Martin, 2015). MuD is implicated in TRAIL-induced apoptosis signaling (Lee et al., 2008; Shin et al., 2013; Choi et al., 2016). Studies have shown BID and Bcl2 as molecules acting upstream and downstream of MuD, respectively (Choi et al., 2016), suggesting that MuD may perform a novel role in the cancer apoptotic pathway. Our analysis identified several candidate genes, including *EXOC5*, *PPP2R5E*, and *SOS2*, and showed that *PPP2R5E* and *SOS2* expression levels correlated with MuD level in tumor cells to some extent. We failed to detect any correlation between *MuD* and *EXOC5* expression; however, *EXOC5* is adjacent to *MuD* (*EXOC5* 5′-UTR starts at Chr14: 57,268,899 and *MuD* 5′-UTR starts at Chr14: 57,268,888). As *MuD* KO or stable expression showed no effect on *EXOC5* expression, there is a possibility that the correlation between *EXOC5* and *MuD* expression may be related to the positional effect. A previous study using a KO mouse model showed that the deletion of *EXOC5* led to apoptosis and disorganization of hair cell stereocilia bundles (Lee et al., 2018). *PPPR2RE* is known as a tumor suppressor gene, and its downregulation induces growth inhibition and apoptosis in gastric cancer cells (Liu et al., 2014). *SOS2* encodes a Ras-specific guanine nucleotide exchange factor (Esteban et al., 2000), and its downregulation decreases the level of Ras and activation of MAP kinase kinase1/2 (MEKK1/2), ultimately inhibiting TNFα-induced apoptosis (Kurada et al., 2009). Not only *PPP2R5E* and *SOS2* expression levels slightly correlated with that of *MuD* in GBM cell lines but also high expression of these genes was associated with longer survival among patients with proneural GBM at a moderate level (**Supplementary Figure 7**). Further study to validate the involvement of these genes in tumor generation linked to *MuD* is in progress.

Based on the database analyses, we propose that *MuD* expression may be upregulated in ESCA and downregulated in KIRC. Further studies should carefully validate these results to evaluate *MuD* as a biomarker with a putative prognostic role. In addition, *MuD* may play a role in the survival of patients with proneural GBM and could be linked to candidate gene regulation. Taken together, our study suggests a novel role for *MuD* in cancer.

## Data Availability

The datasets generated for this study are available on request to the corresponding author.

## Ethics Statement

The biospecimens utilized in the present study were provided by the Ajou Human Bio-Resource Bank (Suwon, Korea), a member of the National Biobank of Korea, which is supported by the Ministry of Health and Welfare. All samples derived from the National Biobank of Korea were obtained with informed consent under institutional review board-approved protocols. The patients/participants provided their written informed consent to participate in this study.

## Author Contributions

J-WO and JS conceived, designed and wrote the manuscript. Computational analysis was performed by JS. J-HC and JS performed biospecimen analysis. Samples were diagnosed and contributed by JA and S-HK. All authors mentioned above and SJ, JO, D-YY, MR critically reviewed and approved the final version of the manuscript.

## Funding

This research was supported by the Basic Science Research Program through the National Research Foundation of Korea (NRF) funded by the Ministry of Education, Science and Technology (NRF-S201801S00057 (JWO); NRF-2016R1D1A1B03935382 (JWO); NRF-S201806S00067 (JS)).

## Conflict of Interest Statement

The authors declare that the research was conducted in the absence of any commercial or financial relationships that could be construed as a potential conflict of interest.
