# Supplementary material for: MUDENG Expression Profiling in Cohorts and Brain Tumor Biospecimens to Evaluate Its Role in Cancer
Source: Front Genet. 2019 Sep 19;10:884. doi: 10.3389/fgene.2019.00884 (PMC6763691; doi:10.3389/fgene.2019.00884)
Supplement: Supplementary file 1 [file Presentation_1.pdf]

Supplementary Figure 1. Tumor purity frequency histogram and boxplot of MuD expression in normal versus tumor tissue in GBM **(A)** KIRC **(B)**.

(A)

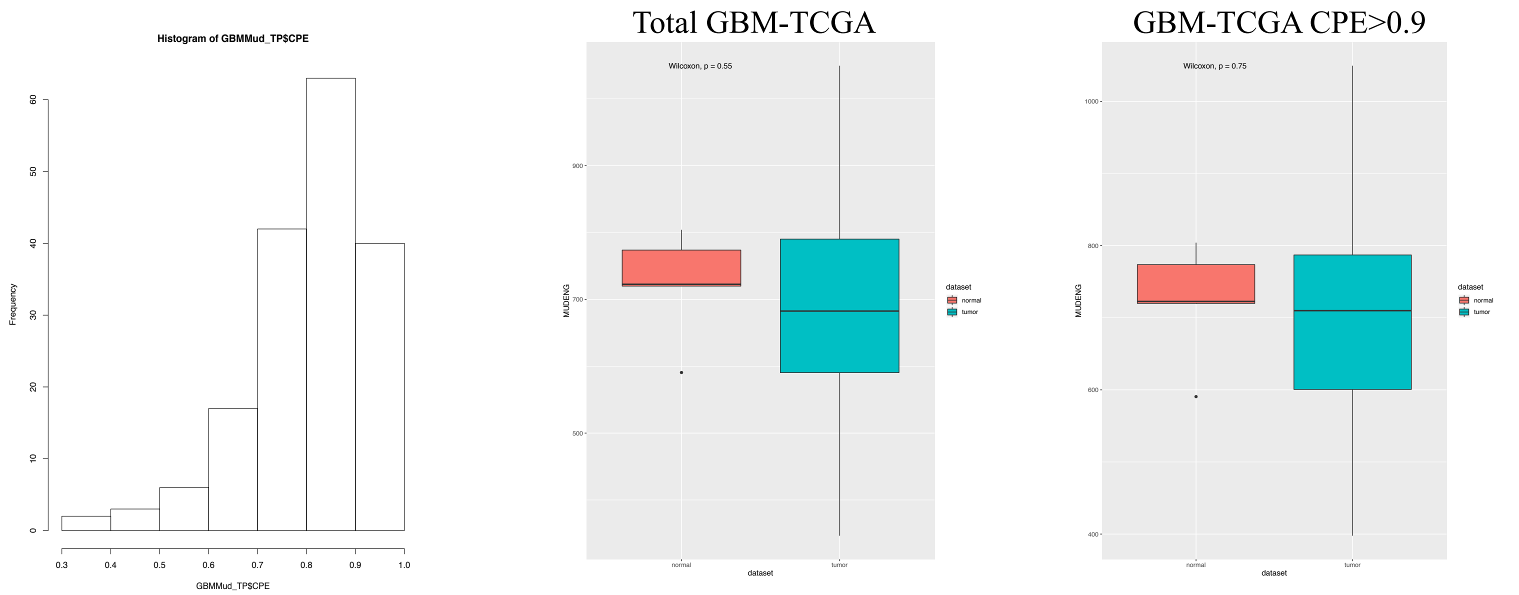

(B)

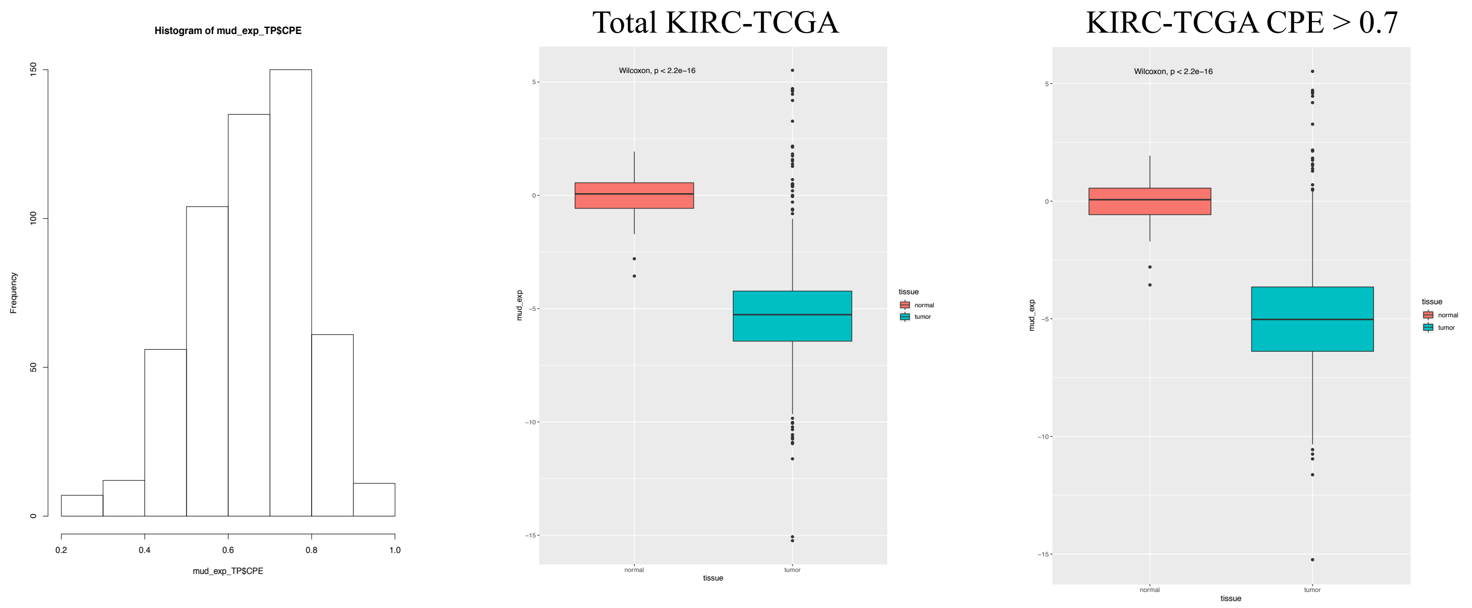

Supplementary Figure 2. Expression of MuD in ESCA based on E-GEOD-23400 as boxplot.

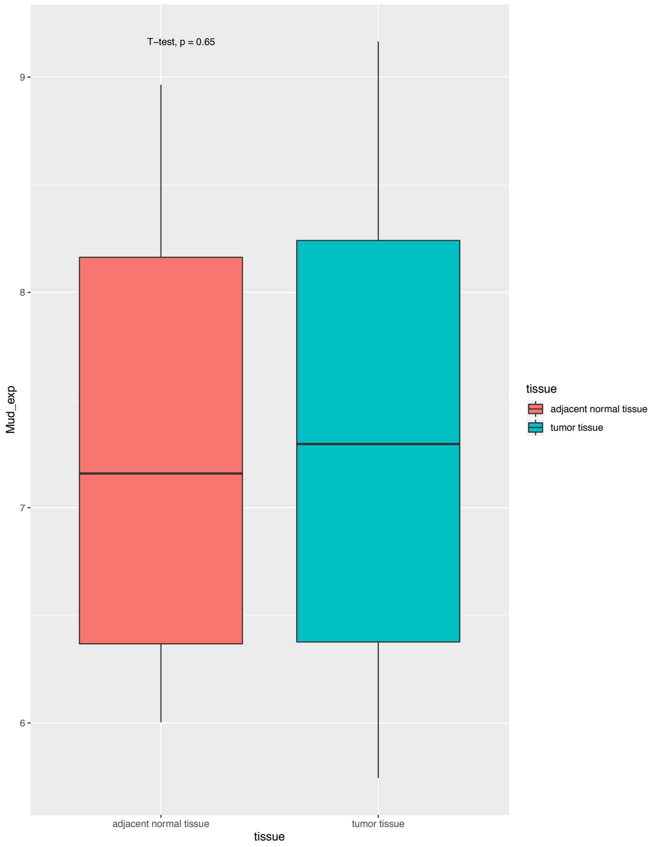

Supplementary Figure 3. Expression of MuD in selected cancer types from TCGA and GTEx database according to GEPIA as box plot. Gene expression at y axis is represented as log2 (TPM +1). X axis represents cancer/tissue types. Gray box indicates MuD expression in normal tissues and red in cancer tissues. TMP=transcripts per kilobase million. * p < 0.005.

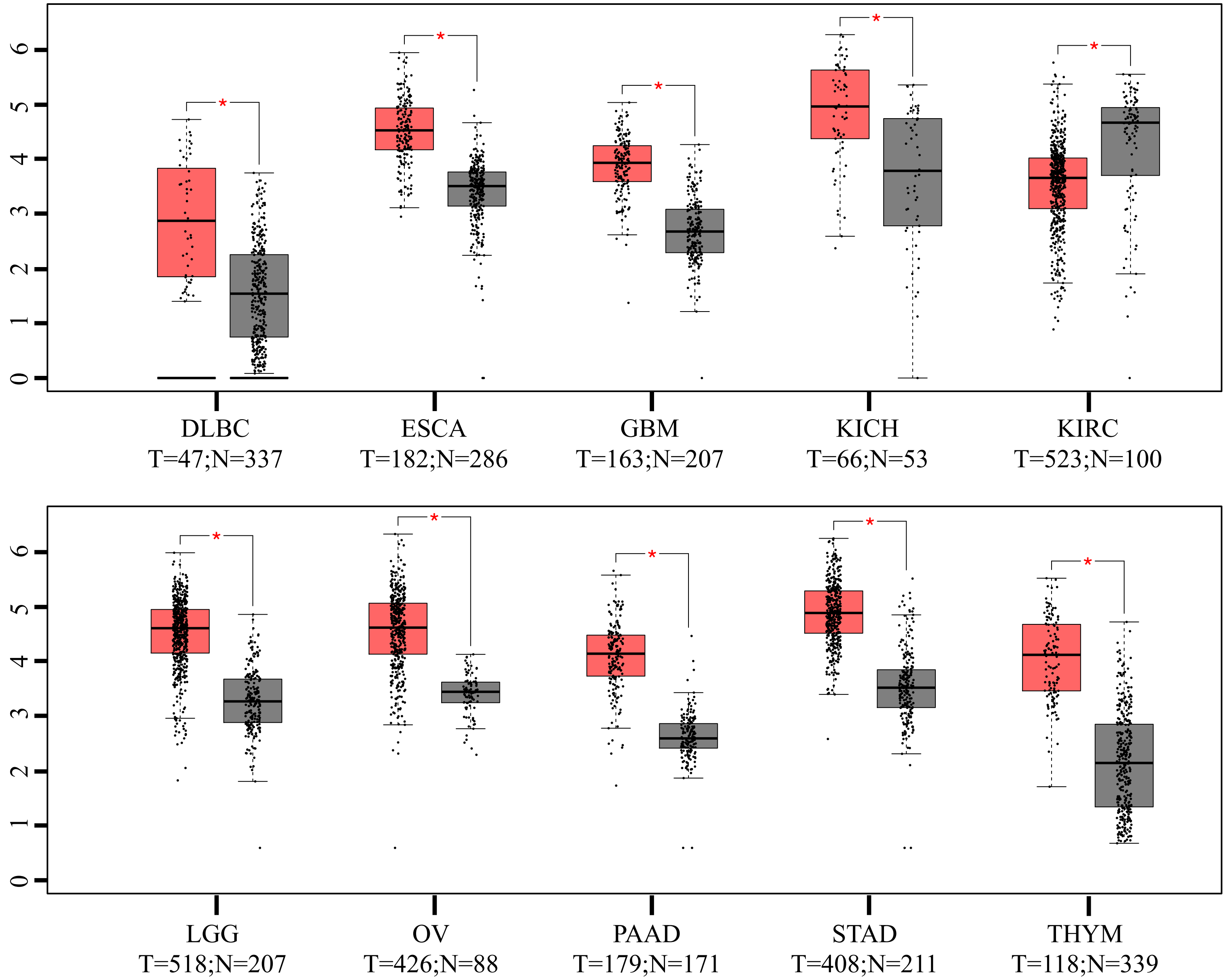

Supplementary Figure 4. Cox proportional analysis of GBM patients according to each subtypes and expression correlation normalized for GBM tumors for EXOC5(Exocyst Complex Component 5), PPP2R5E(Protein Phosphatase 2 Regulatory Subunit B'Epsilon) and SOS2(SOS Ras/Rho Guanine Nucleotide Exchange Factor 2).

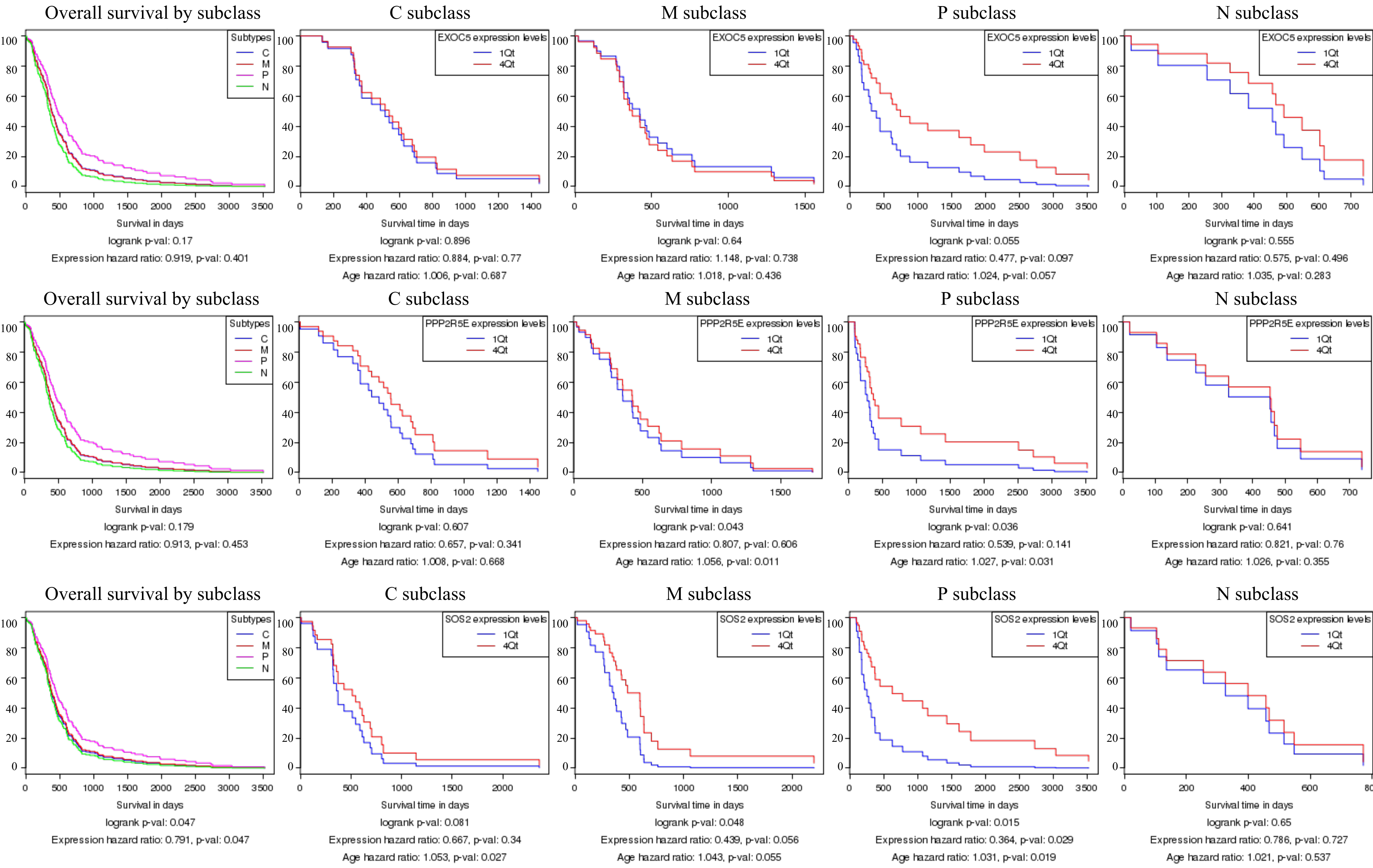

Supplementary Figure 5. Expression of EXOC5, SOS2 and PPP2R5E in 9 GBM tumors, 4 normal brains and 2 astrocytoma biospecimens analyzed by RT-qPCR, normalized to GAPDH and plotted to MuD expression.

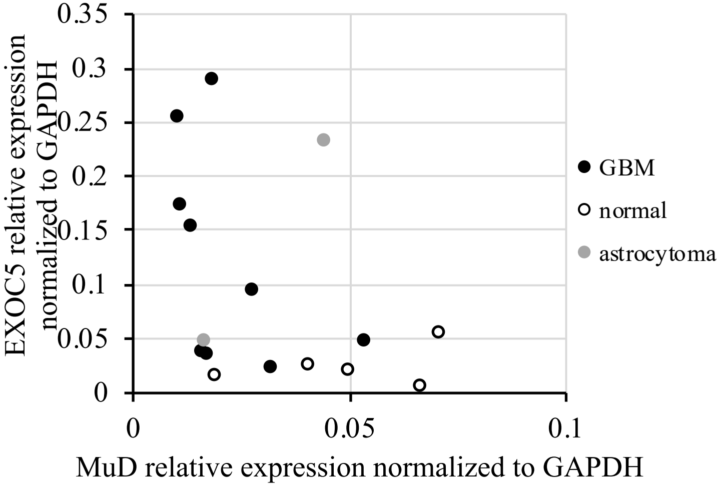

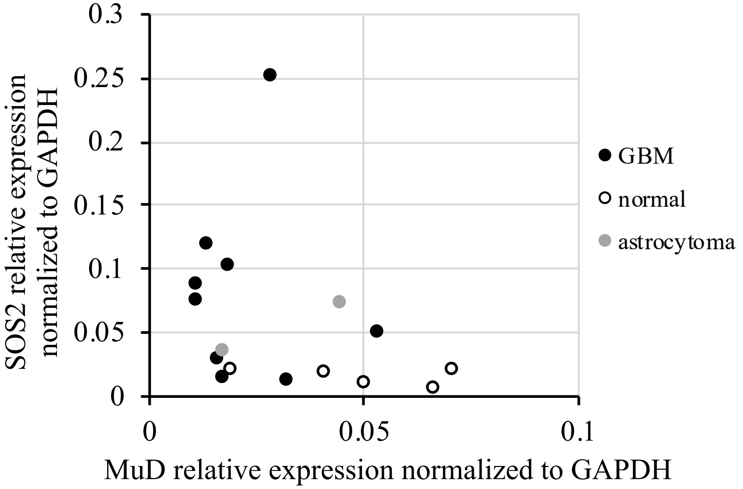

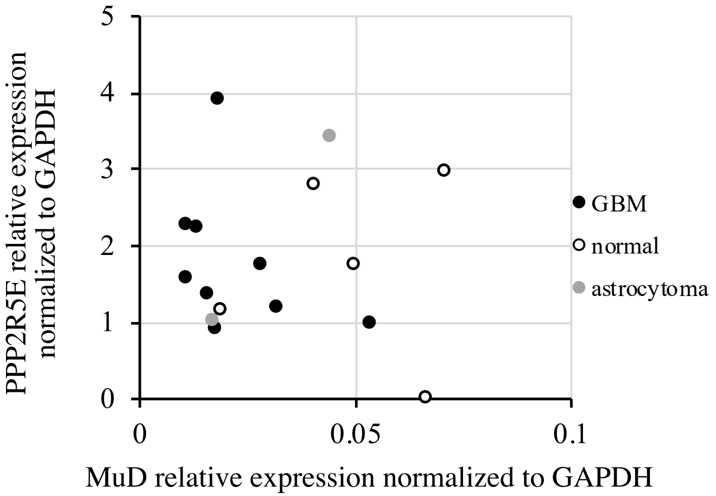

Supplementary Figure 6. Cox proportional analysis of GBM patients according to each subtype for known MuD interacting proteins.

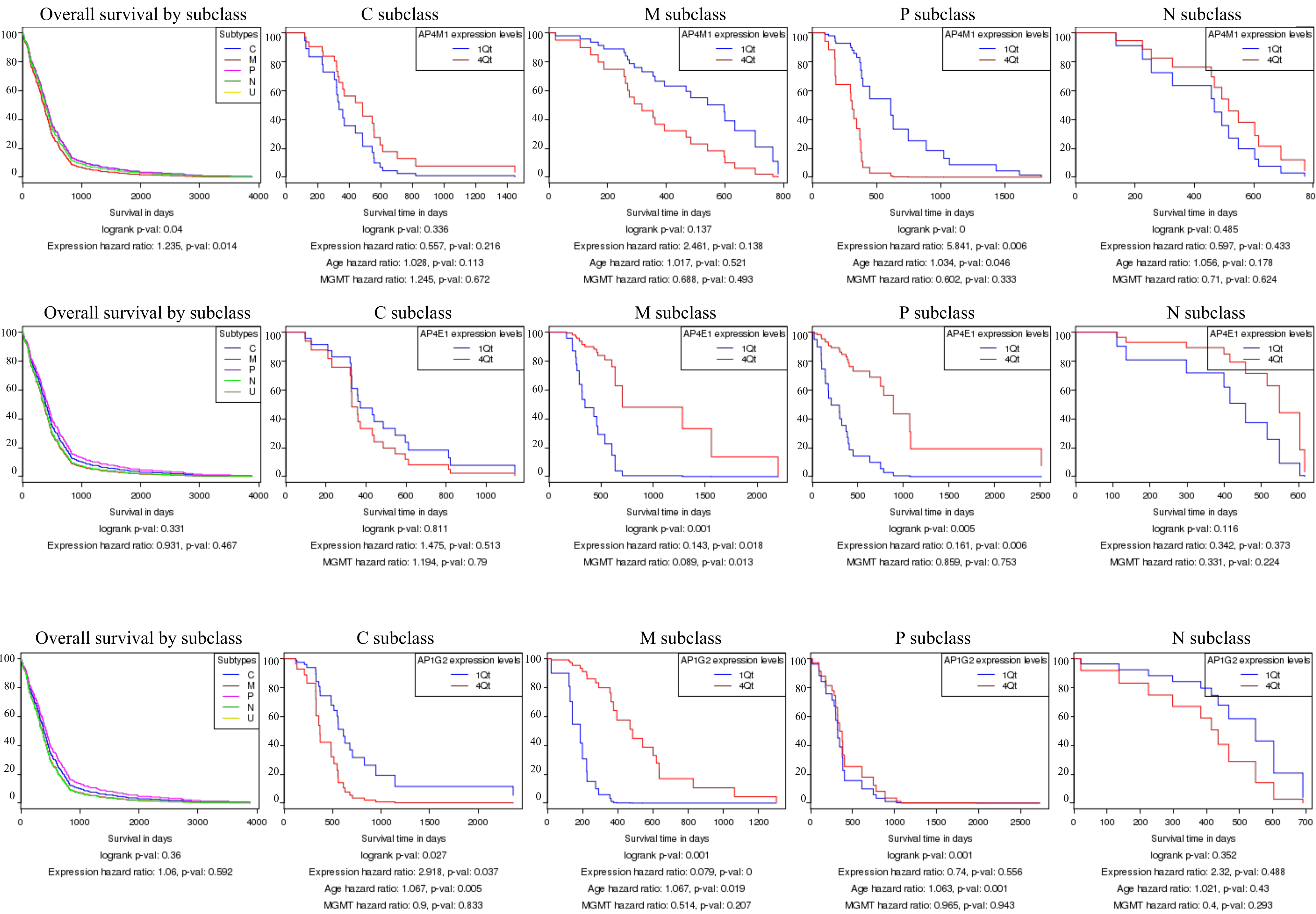

Supplementary Figure 7. Cox proportional analysis of GBM patients according to each subtypes for known MuD interacting proteins (cont).

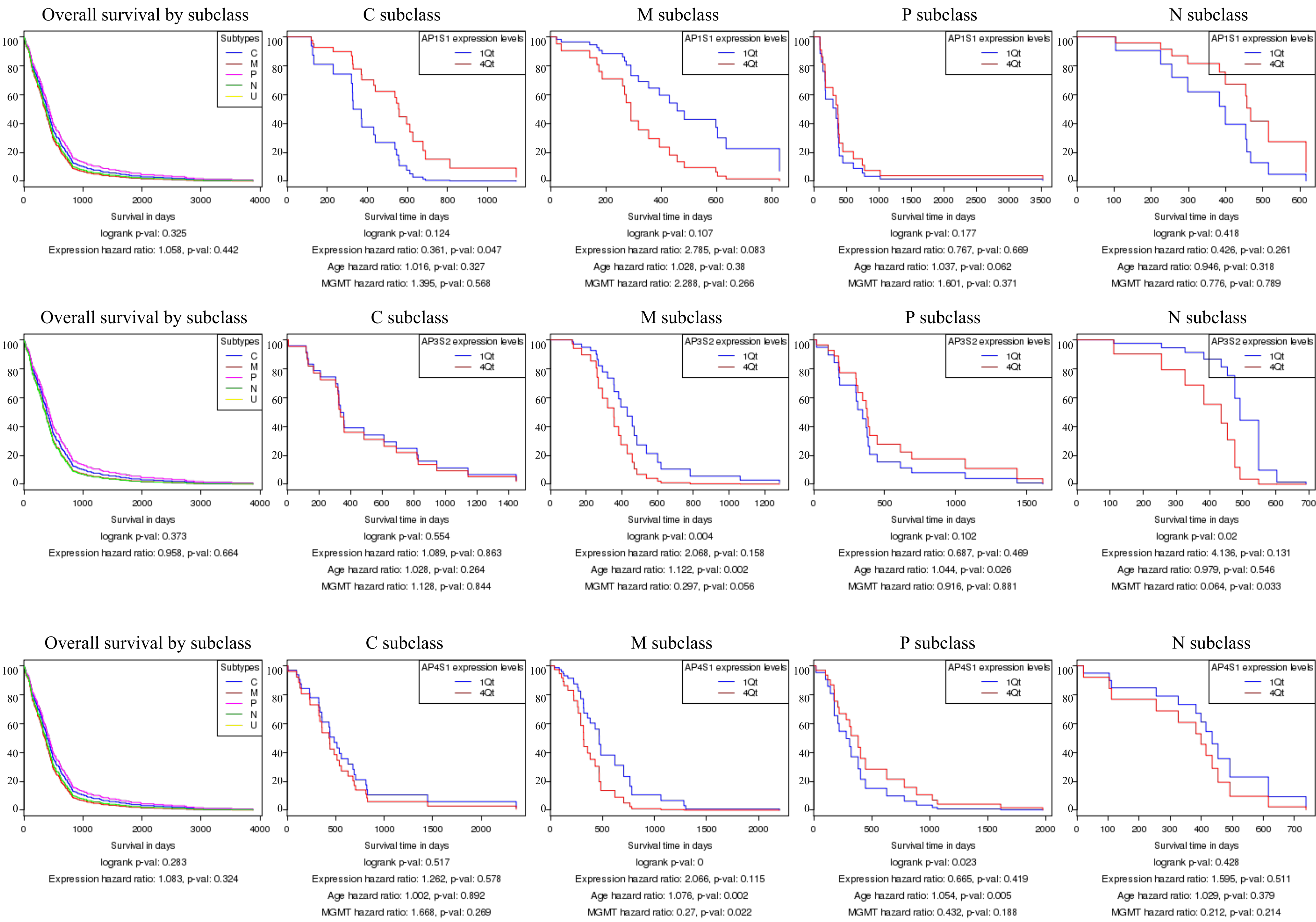

Supplementary Table 1. Top 10 most related genes with MuD expression with Pearson coeffient in BRCA, KIRC, LUAD, ESCA and CHOL according to UALCAN analysis

BRCA		KIRC		LUAD		ESCA		GBM		CHOL	
Genes	PearsonCC	Genes	PearsonCC	Genes	PearsonCC	Genes	PearsonCC	Genes	PearsonCC	Genes	PearsonCC
EXOC5	0.82	PPM1A	0.86	EXOC5	0.81	NAA30	0.74	EXOC5	0.86	SYNJ2BP	0.81
NAA30	0.8	TMEM33	0.84	KIAA0586	0.76	SOCS4	0.72	PPP2R5E	0.85	DLST	0.79
SOCS4	0.79	SYNJ2BP	0.82	SOCS4	0.75	SLC39A9	0.71	NAA30	0.82	C14orf167	0.78
KIAA0586	0.73	NAA30	0.81	PPM1A	0.71	PAPOLA	0.7	SOS2	0.8	EXOC5	0.77
GMFB	0.73	RDH11	0.8	SYNJ2BP	0.69	C14orf101	0.69	ARID4A	0.79	HECTD1	0.76
PPP2R5E	0.72	ATP6V1D	0.8	KIAA0317	0.68	PPM1A	0.68	SOCS4	0.78	SOCS4	0.75
C14orf135	0.71	PDE12	0.79	EXD2	0.67	SMEK1	0.68	C14orf135	0.78	ATP5S	0.75
FBXO34	0.71	SC5DL	0.79	ACTR10	0.67	KIAA0586	0.67	PSEN1	0.77	SCFD1	0.75
ACTR10	0.7	SACM1L	0.79	GMFB	0.67	EXOC5	0.67	PAPOLA	0.77	PPM1A	0.74
ARID4A	0.69	STRBP	0.79	JKAMP	0.66	CPSF2	0.66	MPP5	0.77	COQ6	0.74
